# Factors affecting Baker cyst volume, with emphasis on cartilage lesion degree and effusion in the young and middle-aged population

**DOI:** 10.1186/s12891-021-04721-8

**Published:** 2021-10-05

**Authors:** Murat Saylik, Kemal Gokkus, M. S. Sahin

**Affiliations:** 1 Department of Orthopaedic Surgery, Istinye University Medical Faculty, Topkapı Kampüsü, Maltepe Mah, Edirne Çırpıcı Yolu, No.9 Zeytinburnu, 34010 İstanbul, Turkey; 2 Department of Orthopaedics and Traumatology, Baskent University Alanya Research and Practice Center, Saray Mahallesi Yunus Emre Caddesi No:1 07400, Alanya, Antalya Turkey

**Keywords:** Cartilage, Baker’s cyst volume, Knee arthroscopy, Chondral lesion, Popliteal cyst

## Abstract

**Background:**

The principal aim of this study was to investigate the presence of factors affecting Baker’s cyst volume in young and middle-aged populations.

**Methods:**

Open cyst excision with valve and capsule repair, as well as knee arthroscopy, were used to treat eighty-five patients. The cases were categorized in terms of age, effusion, chondral lesion degree, meniscal tear degree, and Lindgren scores. An ultrasonography (USG) device was used to calculate the cyst volume. The IBM-SPSS 22 program was used for statistical analysis and to assess the relationships between variables using Spearman’s correlation tests.

**Results:**

The degree of chondral lesion was moderately and positively correlated with cyst volume in the total population (correlation coefficient: 0.469; p < 0.05).

The degree of the chondral lesion was moderately and positively correlated with the degree of effusion (correlation coefficient: 0.492; p < 0.005). The cyst volume was weakly and positively correlated with the degree of effusion (correlation coefficient: 0.20; the correlation was at the limits of statistical significance p = 0.07 < 0.08).

**Conclusions:**

This study revealed that an increase in chondral lesion severity increases the amount of effusion and cyst volume.

## Introduction

Generally, joint pathologies, such as chondral lesions and meniscal tears, cause effusion, which leads to an increase in the cyst (Baker) volume [1]. A cystic mass with a large effusion within the popliteal region of the knee was first reported by Guillaume Dupuytren in 1829 [2]. In 1840, Robert Adams explained the correlation between rheumatoid arthritis and swelling of the cystic mass [3]. Anatomical dissection studies determined the mass as the distension of the bursa located between the semi-membranous tendon and the medial head of the gastrocnemius muscle [4–6]. In 1856, Foucher also reported the case of a recurrent cyst that became firm with full knee extension and softer with flexion, which led to the conditions being subsequently coined “Foucher’s Sign” [7].

In 1877, Baker confirmed the entity as a “bursal distention” caused by the trapping of fluid in a bursa with a direct relation to the semi-membranous tendon. Moreover, he explained that the communication between the cyst and the joint synovium behaves as a one-way valve, with fluid leaking into the bursa and no possible flow in the reverse direction. Baker defined the possibility of a ruptured bursa resembling venous thrombosis. For that purpose, Baker’s name was incorporated with the clinical entity of a popliteal cyst, that is, “Baker’s cyst” [8, 9].

Baker’s cyst is strongly associated with the existence of intra-articular pathologies, such as chondral lesions and meniscal tears, with a reported incidence of approximately 94% in some series [10]. Any pathology that causes significant and persistent joint effusion can lead to Baker’s cyst [11]; however, this will depend not only on the presence of the pathology, but also on its extent and severity. The literature can be divided into two groups based on this: the first group of studies correlates the extent of pathologies with Baker’s cyst [12–14], while the other group correlates only with the presence and absence of pathologies [15–22].

However, the quantification of cyst size (calculating the approximate volume) and a probable relationship between cartilage lesion degree, effusion degree, and cyst volume were only reported in one study by Balik et al. [13]. In their study, Rupp et al. [21] reported that articular cartilage lesions were most often associated with a popliteal cyst, whereas Cao et al. [20] found that popliteal cyst or sub-gastrocnemius bursitis were significantly and positively associated with knee cartilage defects at the medial and lateral tibiofemoral sites, before and after adjustment for age, sex, BMI, disease status, and knee radiographic features.

The main objective of this study was to assess the factors affecting Baker’s cyst volume, its importance on effusion, and the degree of cartilage damage in the young and middle-aged population.

## Material and method

This retrospective study was approved by the ethics committee of our institution and was registered under the protocol.

The present study was conducted in accordance with a recognized international standard, including the principles of the Declaration of Helsinki.

Data from 164 patients with Baker’s cysts ≥3 cm in diameter were collected between January 2009 and December 2017. In the current study, cysts with a diameter of ≥3 cm were included, because we assumed that 3 cm was the asymptomatic limit, referring to the study by Tschirch et al. [23]. Screening included all patients with a popliteal mass or mass-like symptoms, including pain in the popliteal fossa or various degrees of joint limitation, consistent with the physical findings of a probable Baker’s cyst. All information was obtained using ultrasound (USG) and magnetic resonance imaging (MRI). Eighteen patients with seronegative and seropositive arthritis were excluded from this study. All remaining 146 patients received conservative treatment for approximately six months before the decision to proceed with surgical treatment was made. Conservative treatment included the application of ice, rest, and the use of non-steroidal anti-inflammatory drugs. In our study group, improvement in symptomology was achieved with conservative treatment in 17 patients, and another 17 patients improved with the use of intra-cystic injections. Five patients were lost during follow-up, with the remaining 112 patients (equating to a total of 114 knees) resisting conservative treatment. Patients with osteophytes and advanced joint narrowing indicative of mild to severe osteoarthritis identified by direct radiography and classified according to the Kellgren–Lawrence scale, as well as those with Kellgren–Lawrence grade 3–4 osteoarthritis, were excluded from the study to avoid bias resulting from likely age-related osteoarthritis. (27 patients, 29 knees). In addition, the patients (young and middle-aged) who had chondral lesions resulting from a sprain or any other trauma to the knee were also included in the current study.

Finally, 85 patients (53 women and 32 men) who were treated with posterior open cystectomy with valve and repair of the posterior capsule, in addition to arthroscopic treatment of intra-articular lesions, were included in the study.

**Fig. 1:**
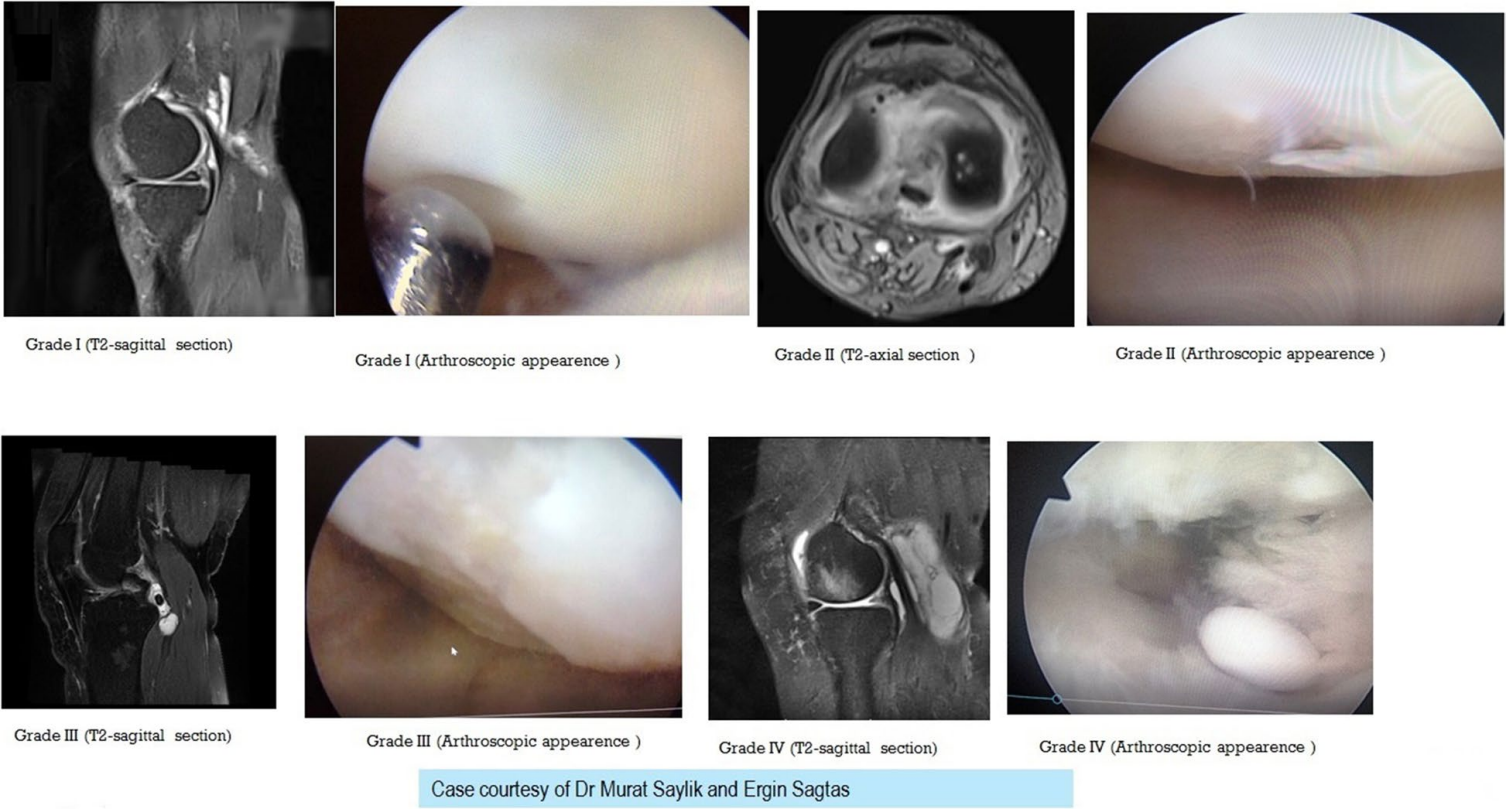
Depicts the grade 1,2,3,4 chondral lesions according to the the outerbridge classification on MRI and arthroscopic images together

**Fig. 2:**
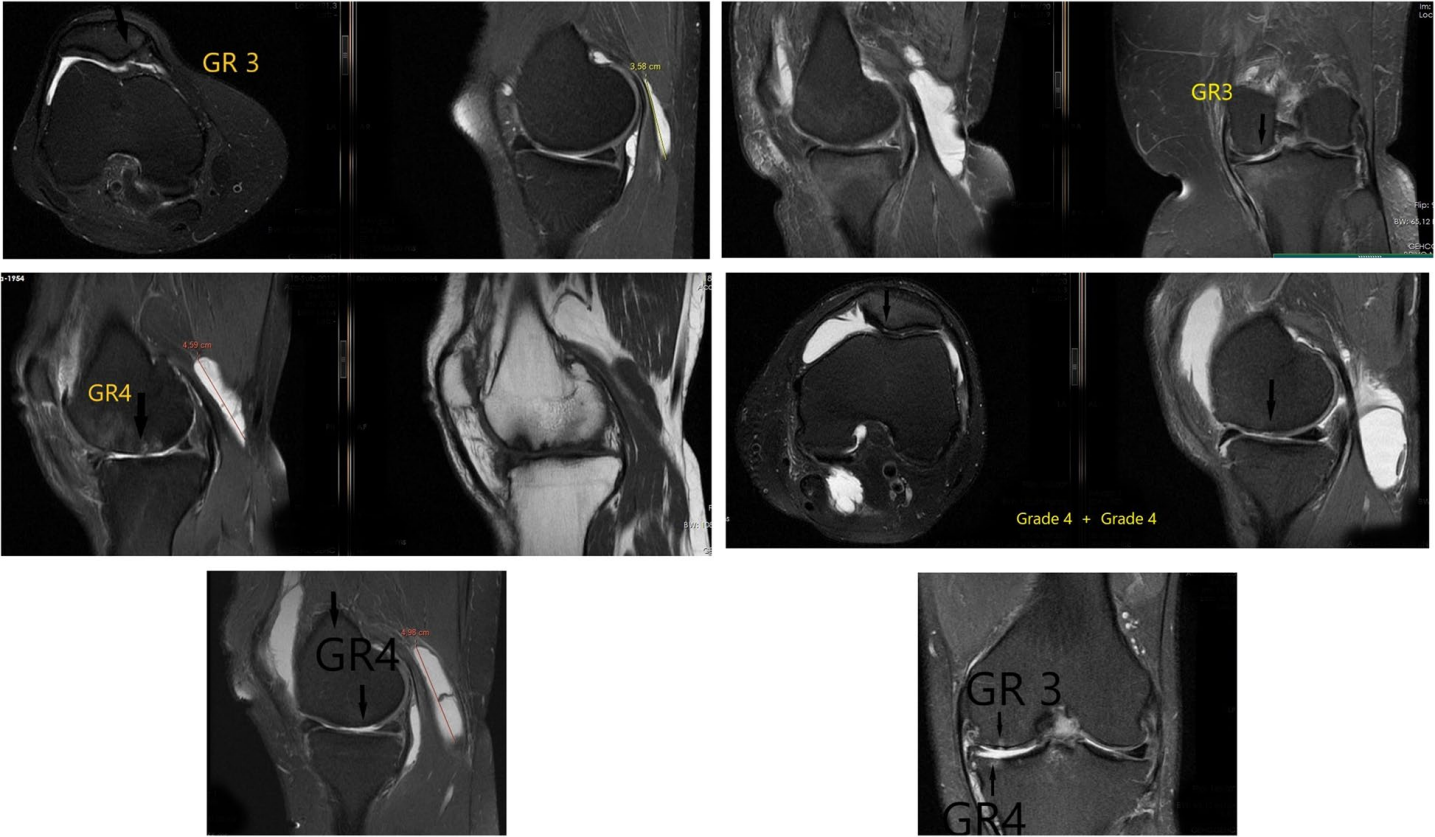
The detailed MRI images of grade 3 and 4 lesions according to the the outerbridge classification

The patients were categorized according to age, sex, effusion level, chondral lesion degree, meniscal tear degree, and Rauschning–Lindgren scores (Table 1). The Rauschning–Lindgren functional score is shown in Table 2. The mean age of the total population was determined to be 46.9 ± 9.2, from a range of 22 to 59 (See Table 3), the chondral lesion degree was graded by applying the modified Outerbridge [24] classification (See Figs. 1 and 2 and Table 4), and the graded results were confirmed with arthroscopy (See Fig. 1).

**Table 1:** For analysis, cases were categorized in age, sex, effusion, chondral lesion degree, meniscal tear degree, and the Rauschning-Lindgren knee scores

Lindgren score value change &distribution of the patients regarding to age , chondral lesion, effusion and meniscal degree									
	Age	Gender	Meniscal tear degree	Chondral lesion degree	Effusion degree	Three dimensions of cyst	Cyst Volume (cm ^**3**^)	Lindgren pre op	Lindgren postop
**1**	49	F	2	5	moderate	5X3X3	23,535	3	1
**2**	53	F	2	1	minimal	4X2,5X1.8	9.414	1	0
**3**	44	M	0	4	moderate	6X3X4	37.656	2	0
**4**	43	F	2	0	minimal	4X2,5X3	15.69	1	0
**5**	44	F	3	5	severe	5,5X2,5X1,1	7.91	3	1
**6**	53	F	2	0	moderate	5X3X2,5	19.61	2	0
**7**	53	M	2	0	minimal	3,5X2X3	10.983	1	0
**8**	58	M	3	2	severe	3X2X1,5	4.707	2	0
**9**	45	F	2	2	minimal	4X2X2,5	***10.46***	1	0
**10**	54	F	2	5	severe	7.2X3,5X2,5	32.949	2	1
**11**	34	F	2	2	minimal	5.2X2X3	16.317	1	0
**12**	50	F	2	3	severe	3,5X1,5X2	5,491	2	1
**13**	50	F	2	2	moderate	4,5X3X2,5	17.651	2	0
**14**	31	F	0	3	moderate	3,5X2X3	10.983	2	1
**15**	34	M	1	2	moderate	5X1.7X2	8.891	2	0
**16**	56	M	1	3	severe	5X1.6X2	8,368	3	1
**17**	46	M	1	2	moderate	3,5X2X1,5	5.491	1	0
**18**	47	F	1	3	minimal	3X2X6	18.828	2	0
**19**	44	M	0	4	moderate	4.2X2X1,5	6.589	2	0
**20**	31	F	1	3	minimal	3.2X2,5X2	8.368	1	0
**21**	39	F	0	3	moderate	5.8X3.7X2.3	25,814	2	1
**22**	54	M	1	4	severe	4X2,5x**2,1**	10.983	2	0
**23**	33	M	2	3	moderate	3.8X2X1.8	7.154	2	0
**24**	50	M	2	4	severe	6X2,5X1,5	11.767	2	1
**25**	30	F	2	1	minimal	4,5X3X1,5	10.59	1	0
**26**	43	F	0	2	minimal	4,5X3X2	14.121	1	0
**27**	22	M	2	0	severe	4X3X2,5	15.69	1	0
**28**	51	F	2	4	severe	4X2,5X2	10.46	2	1
**29**	57	F	2	4	moderate	6.2X2,5X3,5	28,372	2	1
**30**	44	F	1	**7**	severe	10,5X2,5X3	41.186	3	0
**31**	49	F	1	2	minimal	3,5X2,5X3	13.728	2	0
**32**	31	M	2	2	minimal	3,5X4,5X2	16.474	1	0
**33**	53	F	1	2	minimal	6X1,5X2	9.414	1	0
**34**	57	F	2	**8**	severe	8,5X4X3	53.346	2	1
**35**	59	F	1	3	minimal	3,5X4X4,5	32.949	1	0
**36**	43	M	1	4	minimal	3,5X4,5X3	24.711	2	1
**37**	42	F	0	**7**	severe	9X3,5X2,5	41.186	1	0
**38**	28	M	2	2	minimal	3X5X2	15.69	1	0
**39**	59	F	2	4	severe	6X1,5X2,5	11.767	2	1
**40**	56	F	1	3	moderate	5X4X2,5	26.15	2	1
**41**	41	M	2	2	minimal	3,5X2x2,5	9.152	2	0
**42**	45	F	2	4	severe	6X3,5X1	10.983	2	1
**43**	49	M	0	2	minimal	5X3X2,5	19.61	1	0
**44**	59	M	3	4	minimal	5X2,48x**2,3**	**14.915**	3	1
**45**	58	M	2	2	minimal	3,5X1.7X1,5	4.66	2	0
**46**	50	F	2	2	minimal	3,5X2.8X2.3	11.788	1	0
**47**	26	F	0	0	minimal	3X3.2X2.2	11.045	1	0
**48**	58	F	3	**6**	severe	3,5X7X2,5	32.03	3	1
**49**	38	M	2	2	minimal	3X3X2,5	11.767	1	0
**50**	54	M	1	4	minimal	6X3X1	9.41	3	1
**51**	43	F	1	**8**	moderate	12X5X2,5	78.45	2	0
**52**	44	M	2	2	severe	8,5X4,5X1,5	30.007	1	0
**53**	56	M	1	2	moderate	5X2x2	10.46	1	0
**54**	43	M	2	0	minimal	5X1,5x**1,2**	4.707	1	0
**55**	37	M	2	0	minimal	3X6,5X2	20.39	1	0
**56**	34	M	2	2	minimal	6,5X2,5X2	16.997	1	0
**57**	53	M	2	**7**	severe	8,5X4X3	53.346	2	0
**58**	48	F	2	2	minimal	3X6X2,5	23.535	3	1
**59**	48	M	2	0	moderate	3.2X2.8X1,5	7.029	1	0
**60**	31	M	2	3	moderate	4,5X1.7X3	12.002	2	0
**61**	48	F	2	2	minimal	5X3X2	15.69	1	0
**62**	58	F	2	2	minimal	6.1X2X1.7	10.847	3	0
**63**	58	F	3	5	severe	3.1X6.9x**2,1**	**23.492**	3	1
**64**	54	F	2	**3**	moderate	6.1X2.6x **2,3**	**19.077**	2	0
**65**	27	F	2	0	moderate	4.2X1.8x**1,8**	7.116	1	0
**66**	50	F	1	4	minimal	6,5X3X2	20.397	2	0
**67**	35	M	2	0	minimal	5X1.2x**1,1**	3.451	1	0
**68**	53	F	2	5	severe	5.9X2,5x**2,4**	**18.51**	2	1
**69**	54	F	1	3	severe	6X2,5x**1,5**	**11.767**	3	2
**70**	47	F	2	0	minimal	7.8X2x**1,1**	**8.974**	1	0
**71**	58	F	2	2	severe	8.4X1.3x1,2	6.853	1	0
**72**	57	F	2	2	minimal	5.7X2,5x2	**14,905**	2	1
**73**	50	F	2	3	severe	8,5X4X**1,2**	**21.338**	2	1
**74**	49	F	2	2	severe	4,5X2x**1,9**	**8.943**	1	0
**75**	41	F	0	2	moderate	7,5X2x**2**	**15.69**	1	0
**76**	51	F	0	3	severe	5X2,8x2,7	**19.769**	2	0
**77**	47	M	2	0	minimal	5X3x**2,1**	16.474	1	0
**78**	58	F	2	2	minimal	5X2x**1,3**	**6, 799**	2	0
**79**	50	F	2	**7**	severe	9X4,5x**2**	42.363	2	0
**80**	50	M	2	4	severe	5X4,5x**2,1**	**24.711**	3	2
**81**	44	F	2	2	moderate	3.2X1,5x**1,4**	**3.514**	1	0
**82**	58	F	0	**4**	minimal	3.6X1.7x**1,6**	**5.121**	2	1
**83**	50	K	2	2	severe	9.2X3x**1,2**	**17,321**	2	0
**84**	48	M	2	0	minimal	5X2.29x*1*	**5.988**	1	0
**85**	54	F	2	4	minimal	8X5x**1,1**	**23.012**	2	0

**Table 2:** Rauschning and Lindgren grading of knee joint symptoms^a^

	Grade 0	Grade 1	Grade 2	Grade 3
Swelling and pain	no swelling or pain	slight swelling and discomfort after strenuous exercise	moderate swelling and pain following moderate exertion	considerable and tense swelling, severe pain interfering with activities of daily living, pain at rest
Range of motion	no limitation of range of motion	some giving-way or weakness, muscular atrophy < 1 cm	limitation of range of motion between 10 and 20 degrees	limitation of range of motion > 20 degrees
Instability and weakness	no instability or weakness	negligible limitation of range of motion (< 10 degrees)	slight or moderate instability, locking, and muscular atrophy 1-2 cm	disabling instability, contractures, and muscular atrophy > 2 cm
The situation at work or sports participation	no limitation in work or sports participation	no hard labor, no elite sports participation	no physical work, limited participation in sports	stopped working due to knee derangement, no participation in sports

^a^ Rauschning W, Lindgren PG. Popliteal cysts (Baker's cysts) in adults. I. Clinical and roentgenological results of operative excision. Acta Orthop Scand. 1979;50(5):583-591

**Table 3:** The average values and standard deviations of meniscal tear, chondral lesion degree, effusion, Cystvolume, age, Lindgren pre-op, post-op, and Lindgren change

Descriptive Statistics				
	Minimum	Maximum	Mean	Std. Deviation
**Meniscal tear**	1,00	4,00	2,58	0,82
**Chondral lesion degree**	1,00	8,00	2,94	1,71
**Effusion**	1,00	3,00	1,88	0,86
**Cyst volume**	3,45	78,45	17,25	12,63
**Age**	22,00	59,00	46,86	9,17
**Lindgren preop**	1,00	3,00	1,73	0,70
**Lindgren postop**	0,00	1,00	0,29	0,46
**Lindgren change**	1,00	3,00	1,40	0,54

**Table 4:** Outerbridge classification

Grade	Description
**Grade 0**	normal cartilage
**Grade 1**	signal intensity alterations with an intact surface of the articular cartilage compared with the surrounding normal cartilage
**Grade 2**	partial-thickness defect of the cartilage
**Grade 3**	fissuring of the cartilage to the level of the subchondral bone
**Grade 4**	exposed subchondral bone

Using MR images, the meniscal tears were classified as normal meniscus, Grade 0, with Grades I and II having an intra-meniscal signal that did not extend to the free edge. Grade III had a signal shift that abutted the meniscal free border, suggesting a meniscal tear [25]. The Lindgren score change and distribution of the patients regarding age, chondral lesion, effusion, and degree of meniscal injury were recorded (See Table 1).

For a linear statistical evaluation, cases with two or more separate chondral lesions, such as combined patellofemoral and femoral medial condyle lesions, were assessed. In other words, a case with a medial femoral condyle Grade 3 lesion and an additional patellar cartilage Grade 2 lesion was recorded as Grade 5, suggesting that each chondral lesion had the potential to generate effusion of its own.

For cyst volume calculation, the cyst radius was measured in three dimensions, and these measurements were entered into the USG device (Samsung HS50TM, South Korea). The device automatically calculates volumes using a standard formula for calculating the volume of ellipsoidal objects [26]. The degree of effusion was graded according to the study by Martí-Bonmatí et al. [14]. Effusion was classified as minimal: Grade 1 (liquid within lateral recesses or the suprapatellar bursa), moderate: Grade 2 (mild effusion, with both the lateral and suprapatellar recesses filled) or severe: Grade 3 (severe effusion associated with marked distension of the joint cavity).

All raw data obtained from the calculations and measurements are listed in Table 1. The average values and standard deviations of meniscal tear and chondral lesion degree, effusion, cyst volume, age, and Lindgren preoperative, postoperative, and Lindgren changes are listed in Table 2.

The IBM-SPSS 22 software was used for the statistical analysis to assess the relationship between variables using Spearman correlation tests, and to determine the strength and direction of these relationships. The Kolmogorov–Smirnov test was used to assess the normality of the distribution of data, whereas the preoperative and postoperative Lindgren values were evaluated using the Wilcoxon signed-rank test. Chondral lesion and cyst volume variables were recorded and analyzed for a possible relationship, where the statistical significance level was set as p < 0.05 Correlations (denoted as “r”) had different strengths [27]. The relationship between the degree of the chondral lesion and cyst volume was evaluated using the Spearman correlation test (total population) (see Table 5).

**Table 5:** Correlations among age, imaging, and arthroscopic findings

Correlations ^**c**^								
			Meniscal tear degree	Chondral lesion degree	Effusion	Cyst Volume	Age	Lindgren change value
Spearman's rho	Meniscal tear degree	Correlation Coefficient	1,00	-0,13	0,09	-0,10	0,19	0,04
		Sig. (2-tailed)		0,22	0,40	0,35	0,08	0,73
	Chondral lesion degree	Correlation Coefficient	-0,13	1,00	0,492^**^	0,469^**^	0,324^**^	0,345^**^
		Sig. (2-tailed)	0,22		0,00	0,00	0,00	0,00
	Effusion	Correlation Coefficient	0,09	0,492^**^	1,00	0,20^***^	0,19	0,11
		Sig. (2-tailed)	0,40	0,00		0,07***	0,08	0,32
	Cyst Volume	Correlation Coefficient	-0,10	0,469^**^	0,20	1,00	0,07	0,13
		Sig. (2-tailed)	0,35	0,00	0,07		0,53	0,24
	Age	Correlation Coefficient	0,19	0,324^**^	0,19	0,07	1,00	0,232^*^
		Sig. (2-tailed)	0,08	0,00	0,08	0,53		0,03
	Lindgren change value	Correlation Coefficient	0,04	0,345^**^	0,11	0,13	0,232^*^	1,00
		Sig. (2-tailed)	0,73	0,00	0,32	0,24	0,03	

(**) Correlation is significant at the 0.01 level (2-tailed)

(*) Correlation is significant at the 0.05 level (2-tailed)

c. Listwise N = 85

(***) The correlation was at the limits of statistical significance (p=0,07<0,08)

r: Correlation coefficient, r= 0.100 to 0.300 = Weak correlation/relationship

r= 0.300 to 0.500 = Moderate (Fair) relationships/Medium correlation

r= 0.600 and above = Strong relationship/high correlation

The relationship between the degree of chondral lesion and age, relationship between the degree of chondral lesion and the change in Lindgren score, relationship between the degree of chondral lesion and grade of effusion, and relationship between the grade of effusion and cyst volume were assessed using the Spearman rank correlation (Spearman's rho) test (see Table 3).

## Results

Over the four-year follow-up period, USG and MRI were performed only in symptomatic patients. Overall, nine patients reported discomfort and pain during the follow-up period, with four patients undergoing USG imaging and four more undergoing MRI. A recurrent cyst was identified in two of these patients, with an overall incidence rate of 2.3%.

The degree of the chondral lesion showed a moderate positive correlation with the cyst volume in the total population (Spearman’s rho, correlation coefficient: 0.469, statistically significant at p = 0.000 < 0.05, see Table 2). The degree of the chondral lesion also showed a moderate positive correlation with age (Spearman’s rho, correlation coefficient: 0.324, statistically significant at p = 0.000 < 0.05). The degree of the chondral lesion showed a moderate positive correlation with Lindgren’s value change (Spearman’s rho, correlation coefficient: 0.345, statistically significant at p = 0.000 < 0.05). The degree of the chondral lesion showed a moderate positive correlation with the degree of effusion (Spearman’s rho, correlation coefficient: 0.492, p < 0.005). The cyst volume showed a weak positive correlation with the effusion degree (Spearman’s rho, correlation coefficient: 0.20, the correlation being at the limits of statistical significance p = 0.07 < 0.08). There was no statistically significant relationship between age and cyst volume (Spearman's rho, p > 0.05). No significant correlation was found between meniscal tear and cyst volume, effusion degree, or change in Lindgren's value (Spearman's rho, p > 0.05). There was no statistically significant relationship between Lindgren value changes and cyst volume, effusion degree, and chondral lesion degree (Spearman's rho, p > 0.05).

## Discussion

The possible correlation between cyst and intra-articular pathologies is a long-debated issue within the medical community. The literature review for the current study revealed that there were many articles on associations or relationships between intra-articular pathologies and Baker's cyst, which led us to divide the results into two groups. The first group included studies on the relationship between the degree of cartilage lesion, the degree of effusion, the degree of meniscal tear, and cyst volume [12–14], while the second group included studies on the association between cartilage lesions, effusion, meniscal tears, and the presence of cysts [15–22] (See Table 6).

**Table 6:** The literature concerning associations/ relations between the intraarticular pathologies and Baker cyst

**The Current Literature that was concerning the relation between the meniscal tear degree, cartilage lesion degree, effusion degree, and cyst volume**								
	The number of subjects	Chondral lesion degree & Cyst size (positive relationship)	Meniscal lesion degree & Cyst size (positive relationship)	Effusion degree & Cyst size (positive relationship)	Plica existence & Cyst size (positive relationship)	Statistical method	Quantification	
Vasilevska et al. [12]	66	+	+	_		Chi-square test and (Mann-Whitney U test	weak	
Balık MS et al. [13]	45	+	_	+	+	Kolmogorov–Smirnov test, Mann–Whitney U test and Kruskal–Wallis test	Strong	
Martí-Bonmatí L, et al. [14]	145	_	+	+	-	Pearson correlation test	weak	
Current study	85	+	_	+	-	Spearman Correlation test	Strong	
**The Current Literature that was concerning the association between the cartilage lesion, effusion, meniscal tear existence, and cyst existence**								
	The number of subjects	Chondral lesion & cyst existence association	Meniscal tear & cyst existence association	Effusion existence & cyst existence association	Plica existence & Cyst size (positive relationship)	Statistical method	Quantification	
Peter Larking [19]	Review article	-	+	-	-	None (Because the article is report or review)	None	
Yuelong Cao et al. [20]	105	+	-	-	-	Multivariate logistic regression	None	
Rupp S, et al. [21]	100	+	-	+	-	Maentel-Haenszel test.	None	
Childress HM (1970) [ 15]	36	-	+	-	-	None (observational study)	None	Early ages (There was no arthroscopy and MRI)
Miller et al. (1996) [18]	400	+	+	+	-	Multivariate logistic regression	None	
Sansone and De Ponti (1999) [17]	46	+	+	-	-	Chi-Square Statistic	None	
Stone KR, et al. (1996) [16]	238	-	+	-	-	None	None	

Childress [15] was one of the first authors to show a strong association between medial meniscal tears and Baker's cysts. In their own evaluation of the MR images of 400 knees, Miller et al. [18] identified a significant relationship between the presence of a Baker’s cyst and knee joint effusion, meniscal tears, and degenerative arthropathy, and accurately reported the coexistence of a Baker's cyst in 80.5% of cases of medial meniscal tear, 31% of cases of anterior cruciate ligament (ACL) rupture, 76.6% of knee joint effusion, and 68% of degenerative knee joint osteoarthritis. Nevertheless, the coexistence of a Baker's cyst with medial plica, medial femoral chondral lesion, or lateral meniscal tear has not been evaluated, nor has the quantitative correlation between cyst volume and the degree of chondral lesion.

Stone et al. [16] and Sansone and De Ponti [17] investigated the association between meniscal tears and Baker's cysts. They found respectively that meniscal tears occurred in 90% and 83% of knees in the presence of a popliteal cyst.

Although the literature has plenty of association reports, the quantification of cyst size, calculating the approximate volume, and a probable relationship between cartilage lesion degree, effusion degree, and cyst volume, were reported in only one study. Rupp et al. [21] reported that articular cartilage lesions are most often associated with popliteal cysts. They suggested that lesions of the articular cartilage play an essential role in the pathogenesis of secondary popliteal cysts. Low-grade chondral lesions and meniscal tears can be treated successfully by arthroscopic means, whereas arthroscopic surgery for high-grade chondral lesions often fails to eliminate effusion. This can be explained by the hypothesis that effusion facilitates fluid leakage through the valve from knee to gastrocnemius-semimembranosus bursa in adult patients.

The current study supports this hypothesis, with evidence of a direct correlation between cyst volume and the degree of chondral lesion. Rupp et al. [21] concluded that the grade of chondral lesions has a more critical effect on the outcome than the presence of meniscal lesions. In cases of degenerative osteoarthritis with grade III or grade IV lesions of the articular cartilage, addressing the intra-articular lesions with arthroscopic surgery to treat the popliteal cyst may be an unrealistic goal. In this study, we believe that it is possible to treat chondral lesions with meticulous evaluation and proper techniques. Cartilage lesions up to grade IV can be treated successfully with careful evaluation and appropriate bone marrow stimulation techniques (debridement, drilling, and microfracture) [28].

Wolfgang Rauschning's classic study [29] and Kim et al. [30] described the etiopathogenesis of popliteal (Baker’s) cysts in detail, with illustrations, including the existence of capsular openings (communication links) between the gastrocnemius-semimembranosus bursa and the knee joint.

The slit-shaped capsular orifice acts as a funnel, opening during knee flexion due to the pulling force of the semimembranosus tendon and closing during extension because of the compressing forces of the overlying tendons.

If a capsular fold and its opening are present, they should be fully resected, and the remnant capsule should be reinforced with solid suturing materials that can withstand pressure changes caused by knee flexion-extension motions.

We believe that treating intra-articular pathologies and closing the valve, in addition to cyst excision, is helpful, and there have been published case series that reported good clinical results of this combination treatment [6].

Peter Larking [19], a senior research advisor, prepared a report in 2011 for the government of New Zealand. Although this article is a report presented to the government, the author widely reviewed the literature and examined the possibility of the coexistence of Baker’s cysts and other knee pathologies. He emphasized the association between effusion, meniscal tear, degenerative arthritis, and Baker’s cyst [19].

Cao et al. [20] found in their cross-sectional study that popliteal cysts or sub-gastrocnemius bursitis were significantly and positively associated with knee cartilage defects at the medial and lateral tibiofemoral sites before and after adjustment for age, sex, BMI, disease status, and knee radiographic features. Moreover, they also reported that, in multivariable analysis, both were significantly associated with medial tibiofemoral bone marrow lesions (BMLs), but not with lateral tibiofemoral BMLs. They graded cartilage defects and investigated the association between the presence of cysts and the degree of cartilage defects [20]. The main difference between this and the current study is that they targeted an older population, namely 50–79-year-olds, and thus did not exclude patients who had probable established osteoarthritis.

Furthermore, the relationship between intra-articular pathologies and cyst volume has been investigated and discussed in the literature (see Table 6). Martí-Bonmatí et al. [14] performed an MRI-based study in which cysts and cartilage lesions were categorized. Cyst volume was classified as absent, minimum, moderate, or massive. In contrast to the current study, cartilage lesions were categorized as either absent, chondropathy, or arthrosis. However, no relationship was reported between cyst volume and cartilage lesions.

In the current study, cartilage lesions were assessed using the Outerbridge classification, and cyst volume was measured using a formula to calculate the volume of ellipsoidal objects.

Initially, the Outerbridge classification was established to assess the degree of patellar cartilage lesions. In the current study, the Outerbridge classification was used because Kumm et al. [31] reported, when they examined the distribution of pathology in their knee MRI study, that cartilage lesions were more commonly seen in the patellar cartilage.

Although the current study had access to a relatively limited number of cases, it was possible to collect more numerical data, which helped to avoid bias and provide more accurate statistical results. In contrast with Martí-Bonmatí et al.’s [14] study, the current study demonstrated a direct and positive relationship between the degree of cartilage lesions and cyst volume.

We also noted that the amount of effusion and cyst volume was reported to be directly and positively related. Vasilevska et al. [12], in a study involving 66 patients, reported that the size of the Baker’s cyst is strongly correlated with degenerative changes in the cartilage and with the degree of meniscus degeneration. However, contrary to both Martí-Bonmatí et al. [14] and Vasilevska et al. [12], the current study results did not show an effect of meniscal tear degree on the amount of effusion and cyst volume. We attribute this outcome to the distribution of meniscal tears in our group, where the majority consisted of Grades 0, 1, and 2 tears, in which the meniscal tears did not reach the joint surface and could not impair joint mechanics to cause effusion. In addition, these two studies did not quantify the Baker’s cyst size by calculating the volume but instead classified their size as either large or small; furthermore, they measured cartilage thickness in the weight-bearing zone and classified cartilage degeneration as total (complete), subtotal, and absent. For our part, we quantified the size of the cyst by calculating the volume, which provided an advantage in statistical and mathematical accuracy. Additionally, our use of the Outerbridge classification with MRI and direct vision arthroscopy enabled us to quantify the degree of cartilage lesions. In the current study, there was a statistically significant correlation between knee effusion and cyst size.

Balik et al. [13] investigated the relationship between Baker’s cyst volume and cartilage degeneration, effusion, and the existence of plica in 45 knees. They concluded that an increase in the degree of cartilage degeneration and the existence of medial plica could cause an increase in intra-articular effusion and cyst volume. This study was remarkably similar to ours and showed parallel results regarding the relationship between effusion, degree of cartilage lesion, and cyst volume. They also showed a similar approach in their results in terms of quantifying cartilage lesions and cyst size. In parallel with the current study, they found no significant correlation between meniscal tear grade and cyst volume. However, we quantified cartilage lesions using the Outerbridge classification and had access to a larger number of knees, which allowed a clearer delineation of the study.

One important distinction from the Balik et al. [13] analysis should be considered, namely the fact that we observed that some patients had more than one cartilage lesion., the combined impact was considered. We believe that this combination should be considered when examining the association between Baker’s cyst volume and cartilage injury. We hypothesized that it would not be incorrect to suggest that knees with multiple chondral lesions may produce more chondral debris than knees with a single chondral lesion.

Another element of the current research that is distinct from the majority of the literature is the chosen patient population. Patients aged >60 years were excluded from the study to avoid any bias associated with age-related osteoarthritis.

In this study, the positive relationship between chondral lesion degree and Lindgren change value should be interpreted as an effective patient therapy, since intra-articular issues, such as chondral injury and meniscal tears, have been carefully handled.

In light of this study, surgeons' decisions in the management of Baker’s cyst associated with the chondral lesion may lean towards cystic excision and the elimination of the chondral lesion.

The association between internal knee derangements (such as meniscal tears and cartilage lesions) and Baker’s cysts is known. However, this study adds new quantitative information regarding the positive correlation between the combined cartilage lesion degree (as a combination of patellar cartilage, femoral and tibial condylar cartilage lesion degrees), effusion degree, and cyst volume in the young and middle-aged population.

The limitations of this study were that the calculation of the cyst volume yields approximate results, that it was retrospective in nature, and that it involved a relatively small number of patients.

Another limitation of the current study was that only symptomatic patients were evaluated postoperatively for recurrence with USG and MRI.

## Conclusion

The current study revealed that the degree of the chondral lesion was significantly correlated with the amount of effusion and cyst volume in young and middle-aged patients with Baker’s cysts. Moreover, the necessity of eliminating chondral lesions in addition to cyst excision in the treatment of Baker’s cysts was also suggested.

## Data Availability

The degree of the chondral lesions and the cyst volumes with cyst diameters (for justification of calculation results) generated or analyzed during this study are included in this published article (see Table 1). The other datasets during and/or analyzed during the current study are available from the corresponding author upon reasonable request.

## Abbreviations

F: Female
M: Male
MRI: Magnetic Resonance Imaging
USG: Ultrasonography
OA: Osteoarthritis
BMI: Body Mass Index
BMLs: Bone Marrow Lesion
ACL: Anterior Cruciate Ligament
